# Next-generation UOWC enabling high-speed and secure RGB image transmission using IRSM-OCDMA with PSO-based image enhancement

**DOI:** 10.1038/s41598-025-30710-1

**Published:** 2025-12-19

**Authors:** Somia A. Abd El-Mottaleb, Ahmad Atieh, Moustafa H. Aly

**Affiliations:** 1 Department of Mechatronics Engineering, Alexandria Higher Institute of Engineering and Technology, Alexandria, Egypt; 2 Optiwave Systems Inc, Ottawa, Canada; 3 Department of Electronics and Communications Engineering, College of Engineering and Technology, Arab Academy for Science, Technology, and Maritime Transport, Alexandria, Egypt; 4 OSA, Washington, USA

**Keywords:** Secure RGB image transmission, Underwater optical wireless communication (UOWC), Identity row shift matrix (IRSM) code, Attenuation and scattering in UOWC, Image quality assessment (IQA) metric, Post-processing median filtering, Particle swarm optimization (PSO), Engineering, Mathematics and computing, Ocean sciences, Optics and photonics

## Abstract

Underwater Optical Wireless Communication systems face severe signal attenuation, scattering, and turbulence, which significantly degrade image transmission quality and limit the communication range. To address these challenges, this paper proposes a secure and high-capacity RGB image transmission framework based on Optical Code Division Multiple Access (OCDMA) using Identity Row Shift Matrix (IRSM) codes. The IRSM-OCDMA scheme enhances data confidentiality by assigning unique orthogonal codes to each user while supporting simultaneous multiuser transmission with an aggregate rate of up to 30 Gbps. System performance is analyzed across five water types: Pure Seawater (PS), Clear Ocean, Coastal Ocean, Harbour I, and Harbour II (HR II), covering a broad range of attenuation coefficients. Image quality is quantitatively evaluated using standard metrics including Root Mean Square Error, Signal-to-Noise Ratio, Peak Signal-to-Noise Ratio, Structural Similarity Index Measure, and Correlation Coefficient. Two distinct post-processing methods are applied: median filtering for impulsive noise reduction and a Particle Swarm Optimization-based correction algorithm that adaptively restores image features under underwater channel conditions. Simulation results show a maximum transmission distance of 27 m in PS and 4 m in turbid HR II water, demonstrating the effectiveness of the proposed framework. The combination of IRSM coding with adaptive post-processing offers a robust solution for secure, high-quality image transmission in Internet of Underwater Things applications.

## Introduction

The increasing demand for real-time data transfer in diverse underwater applications, including oceanographic studies, subsea resource management, ocean environmental monitoring, and robotic deployments, is fueling advancements in high-speed underwater wireless communication systems^1,2^. Traditional underwater communication methods relying on acoustic signals face challenges related to restricted bandwidth and significant delays. These limitations impede the implementation of real-time applications, including remote operation and video transmission from underwater vehicles^3^.

Radio Frequency (RF) waves, on the other hand, were once a standard approach for relaying information from underwater to terrestrial locations. However, because water strongly absorbs radio signals, this method is only viable for very short-range communication, highlighting the need for alternative solutions^4,5^. Thus, conventional RF and acoustic communication systems face significant challenges in meeting the stringent range and bandwidth requirements of underwater data transmission, particularly in both shallow and deep-sea environments. Optical Wireless Communication (OWC) has emerged as a promising alternative to overcome these limitations^6^. Compared to RF-based systems, OWC offers a significantly broader bandwidth, mitigating spectrum congestion while enabling high-speed data transfer. Moreover, its adaptability with free-space optic and fiber optic communication extends applications across terrestrial and non-terrestrial enhancing its potential for diverse communication scenarios^7^.

Underwater Optical Wireless Communication (UOWC), a specific type of OWC, offers a viable and advantageous alternative to traditional methods due to its capacity for high-speed data transmission and extremely low latency^8,9^. UOWC serves as an effective last-mile solution for a wide array of oceanographic applications ranging from seafloor observation and resource exploration to maritime archaeology, seismic monitoring, and real-time multimedia communication between diverse underwater platforms^8^. Given that, recent research has revealed the detrimental impact of the underwater channel. High concentrations of small particles cause significant absorption and scattering, similar to what is seen in the atmosphere due to particles such as dust, water, gases, etc. This limits effective transmission, due to pathloss and geometric losses from scattering to just tens of meters^10,11^. Underwater optical wave propagation is heavily dependent on wavelength. The study in^12^ demonstrated that optical waves within the 450–550 nm spectrum (encompassing blue and green light) undergo significantly diminished attenuation. This effect is chiefly driven by photosynthetic processes in algae, which intensify in coastal marine environments during seasons with elevated temperatures.

The transmission of images in UOWC systems is crucial for supporting key underwater applications, including marine research, environmental surveillance, and Autonomous Underwater Vehicle (AUV) guidance. Unlike optical communication in terrestrial or aerial environments, UOWC encounters distinct challenges such as water turbidity, absorption, and scattering that significantly impair signal integrity. Mitigating these challenges can improve both the reliability and clarity of transmitted images. Advancements in UOWC image transmission have the potential to transform deep-sea exploration, underwater structure assessment, and rapid disaster response efforts, highlighting its crucial role in connecting surface technologies with the vast, largely uncharted underwater domain.

Securing image transmission in UOWC systems is crucial for some applications including military surveillance, confidential environmental monitoring, and underwater infrastructure assessment. The risk of image data interception or tampering poses significant threats to mission-critical operations and ecological evaluations. To address these concerns, encryption techniques like chaotic iteration of dragon fractal shapes (ChDrFr) was utilized^13^. However, encryption alone may not address UOWC-specific issues like multipath fading or bandwidth constraints, necessitating complementary error-correction protocols. In UOWC, OCDMA leverages the blue-green wavelength band (optimal for underwater propagation) to encode images using spectral or temporal codes. OCDMA enhances security by embedding unique orthogonal codes into the optical signals, allowing multiple users to share the same channel securely^14^. Only receivers with matching codes can decode the transmitted data, inherently preventing unauthorized access. OCDMA also combats channel impairments like scattering and turbulence by spreading signals across multiple wavelengths, improving robustness. Additionally, its multi-user capability aligns with the need for collaborative underwater networks, such as swarm AUVs or distributed sensor arrays.

In this study, and to the best of our knowledge, we propose a secure RGB image transmission in UOWC using OCDMA exploiting Identity Row Shift Matrix (IRSM) codes. The seamless integration of security and efficiency underscores OCDMA potential as a foundational technology for safeguarding the future of UOWC systems. In addition to the secure transmission mechanism, a post-processing enhancement stage based on Particle Swarm Optimization (PSO) algorithm is introduced at the receiver to significantly improve image quality. This PSO-based correction algorithm adaptively optimizes a set of restoration parameters including deblurring, gamma correction, histogram matching, and wavelet-based denoising based on structural similarity to a reference image. This adaptive optimization framework enables robust correction of underwater distortions, resulting in superior perceptual clarity and improved image quality metrics across various water types and transmission distances.

### Contribution

The primary contributions of this study are as follows:

- Securing and enhancing RGB image transmission in UOWC systems by employing the IRSM code within the OCDMA framework.
- Evaluating the proposed secure RGB image transmission system using image quality metrics, including Root Mean Square Error (RMSE), Signal-to-Noise Ratio (SNR), Peak Signal-to-Noise Ratio (PSNR), correlation coefficient, and Structural-Similarity-Index-Measure (SSIM) across five types of water bodies.
- Enhancing RGB image quality over extended underwater distances by applying median filtering at the receiver to mitigate signal degradation.
- Improving perceptual and quantitative image quality through the application of a PSO-based correction algorithm that adaptively tunes restoration parameters to optimize structural similarity with reference images.

### Related work

Several earlier studies have explored using different techniques of OCDMA codes in UOWC systems; however, none has specifically addressed OCDMA coded RGB image transmission. In^15^, an UOWC system employing the Fixed Right Shift (FRS) code within the OCDMA framework was proposed. The study evaluated system performance across three channels transmitted in five types of Jerlov waterbodies, demonstrating successful image transmission over underwater distances ranging from 5.15 to 35 m, depending on the optical properties of the water, with a total transmission capacity of 30 Gbps.

Similarly, the work in^14^ investigated the performance of a UOWC system utilizing the Sigma Shift Matrix (SSM) OCDMA code in pure-sea (PS), clear-ocean (CL), coastal-ocean (CS), harbour I (HR I), and harbour II (HR II) environments. The results indicated that the proposed system achieved an overall transmission capacity of 40 Gbps, supporting underwater distances of 5.05 m for PS, 27.5 m for CL, 16 m for CS, 8.2 m for HR I, and 48 m for HR II.

Additionally, there were studies conducted on image transmission in UOWC system. In^16^, the performance of an RGB image transmission system over a UOWC channel was analyzed in PS, CL, CS, HR I, and HR II waters, employing the On–Off Keying (OOK) modulation scheme. The assessment of reconstructed image quality was conducted using SSIM and peak PSNR metrics. However, the study focused on transmitting a single RGB image at a data rate of 2 Gbps. The findings emphasized that water property variations play a crucial role in degrading image quality, thereby restricting the point-to-point UOWC transmission range. Moreover, the study’s relatively low data rate limits its applicability in high-speed image transmission scenarios. Additionally, no security techniques were applied to secure the transmitted images, potentially exposing them to interception risks.

In contrast, the study in^13^ implemented the ChDrFr ciphering technique on grayscale images and evaluated its performance in a UOWC system across five different water types. While the results demonstrated reliable performance at a data rate of 10 Gbps, this transmission rate remains insufficient for high-speed data applications. Furthermore, the study did not consider RGB image transmission, which is crucial for advanced underwater imaging and communication systems.

Table 1 provides a comparative summary between the proposed system and existing UOWC techniques in terms of transmission range, IQA metrics, security methods, enhancement approaches, and achievable data rates.

**Table 1:** Comparison between the proposed system and prior UOWC works.

References	Range	Image/IQA metrics	Security method	Enhancement techniques	Data rate (Gbps)
^13^	22 m in PS, 15 m in CL, 10.5 m in CS, 5.8 m in HR I, and 4 m in HR II	One gray image; PSNR, SNR, and SSIM were used as IQA metrics	ChDrFr encryption technique	Median filter	10
^14^	48 m in PS, 27.5 m in CL, 16 m in CS, 8.2 m in HR I, and 5.05 m in HR II	No images used	OCDMA technique with SSM code	–	40
^15^	35 m, 31 m, 21 m, 12 m, and 5.15 m in Jerlov types I, IA, IB, II, and III, respectively	No images used	OCDMA with FRS code	–	30
^16^	2600 m in PS, 2100 m in CL, 1800 m in CS, 1600 m in HR I, and 1500 m in HR II	One RGB image; PSNR and SSIM were used as IQA metrics	No security technique applied	Wiener and median filters	2
Present work	27 m in PS, 17 m in CL, 12 m in CS, 6.2 m in HR I, and 4 m in HR II	Three RGB images; RMSE, PSNR, SNR, SSIM, and CF were used as IQA metrics	OCDMA with IRSM code	Median filters and PSO algorithm	30

Additionally, another study employed a Convolutional Neural Network (CNN) to classify received data between 64-Quadrature Amplitude Modulation (64-QAM) and 32-Phase Shift Keying (32-PSK) after propagation through UOWC channel^18^. The study in^19^ investigated the performance of UOWC system utilizing LED source, while a comparison between data transmission in UOWC using Laser Diodes (LDs) and Light Emitting Diodes (LEDs) was presented in^20^. The findings demonstrated that using LDs outperforms LEDs in terms of transmission performance. In^21^, the authors implemented a Multiple-Input Multiple-Output (MIMO) technique in UOWC, and the results indicated that MIMO significantly enhances the achievable underwater transmission range.

### Paper organization

The remainder of this paper is organized as follows. Section “OCDMA code and UOWC channel attenuation” provides a detailed construction of the IRSM code and outlines the attenuation calculation of UOWC channel considering absorption and scattering coefficients. Section “Proposed secured RGB image transmission in UOWC using IRSM code” describes the architecture of the proposed secure RGB image transmission system utilizing IRSM coding in a UOWC environment. Section “PSO-based image enhancement framework for UOWC” introduces the PSO-based image enhancement framework, which adaptively optimizes restoration parameters to improve the perceptual quality of received images under various underwater conditions. Section “Simulation results and analysis” displays and discusses the results, analyzing the system performance based on key evaluation metrics. Finally, Section “Conclusion” concludes the paper with the key findings and outlines potential directions for future research.

## OCDMA code and UOWC channel attenuation

In this study, three different RGB images; namely Image 17 (IMG 17), Image 165 (IMG 165), and Image 242 (IMG 242), are transmitted using distinct IRSM code sequences. The impact of absorption and scattering coefficients in five types of water is analyzed for each transmitted image. This subsection presents the construction of the IRSM code and the attenuation calculations for PS, CL, CS, HR I, and HR II environments.

### RSM code construction

The IRSM code is characterized by code length given by $N$, code weight represented by $W$, and system cardinality denoted by $S$^17^. The IRSM code has zero cross-correlation. The code construction of the IRSM is as follows:

*Step 1:* Generate identity matrix of dimension $S\times S$. In this study, we considered $S=3$. Accordingly, the identity matrix will be given as:

1 $${I}_{S\times S}=\left[\begin{array}{ccc}1& 0& 0\\ 0& 1& 0\\ 0& 0& 1\end{array}\right]$$

*Step 2:* Perform two times right shifting to each row in ${I}_{S\times S}$ to generate matrix ${I}_{S\times S}^{1}$ after first time shifting and ${I}_{S\times S}^{2}$ after second time shifting matrix as follows:

2 $${I}_{S\times S}^{1}=\left[\begin{array}{ccc}0& 1& 0\\ 0& 0& 1\\ 1& 0& 0\end{array}\right]$$

3 $${I}_{S\times S}^{2}=\left[\begin{array}{ccc}0& 0& 1\\ 1& 0& 0\\ 0& 1& 0\end{array}\right]$$

*Step 3:* Convert matrices ${I}_{S\times S}$, ${I}_{S\times S}^{1}$, and ${I}_{S\times S}^{2}$ to row vectors as follows

4 $${I}_{S\times S}=\left[\begin{array}{ccc}1& 0& 0\\ 0& 1& 0\\ 0& 0& 1\end{array}\right]\leftrightarrow \begin{array}{c}\left[\begin{array}{ccc}1& 0& 0\end{array}\right]\\ \left[\begin{array}{ccc}0& 1& 0\end{array}\right]\\ \left[\begin{array}{ccc}0& 0& 1\end{array}\right]\end{array}$$

5 $${I}_{S\times S}^{1}=\left[\begin{array}{ccc}0& 1& 0\\ 0& 0& 1\\ 1& 0& 0\end{array}\right]\leftrightarrow \begin{array}{c}\left[\begin{array}{ccc}0& 1& 0\end{array}\right]\\ \left[\begin{array}{ccc}0& 0& 1\end{array}\right]\\ \left[\begin{array}{ccc}1& 0& 0\end{array}\right]\end{array}$$

6 $${I}_{S\times S}^{2}=\left[\begin{array}{ccc}0& 0& 1\\ 1& 0& 0\\ 0& 1& 0\end{array}\right]\leftrightarrow \begin{array}{c}\left[\begin{array}{ccc}0& 0& 1\end{array}\right]\\ \left[\begin{array}{ccc}1& 0& 0\end{array}\right]\\ \left[\begin{array}{ccc}0& 1& 0\end{array}\right]\end{array}$$

*Step 4:* Flatten ${I}_{S\times S}$, ${I}_{S\times S}^{1}$, and ${I}_{S\times S}^{2}$ into one row vector that has size of $1\times {S}^{2}$ by concatenating the three row vectors as follows:

7 $${I}_{S\times S}\leftrightarrow {I}_{1\times {S}^{2}}=\left[\begin{array}{ccc}\begin{array}{ccc}1& 0& 0\end{array}& \begin{array}{ccc}0& 1& 0\end{array}& \begin{array}{ccc}0& 0& 1\end{array}\end{array}\right]$$

8 $${I}_{S\times S}^{1}\leftrightarrow {I}_{1\times {S}^{2}}^{1}=\left[\begin{array}{ccc}\begin{array}{ccc}0& 1& 0\end{array}& \begin{array}{ccc}0& 0& 1\end{array}& \begin{array}{ccc}1& 0& 0\end{array}\end{array}\right]$$

9 $${I}_{S\times S}^{2}\leftrightarrow {I}_{1\times {S}^{2}}^{2}=\left[\begin{array}{ccc}\begin{array}{ccc}0& 0& 1\end{array}& \begin{array}{ccc}1& 0& 0\end{array}& \begin{array}{ccc}0& 1& 0\end{array}\end{array}\right]$$

*Step 5:* Finally, construct the IRSM matrix, the three row vectors ${I}_{1\times {S}^{2}}$, ${I}_{1\times {S}^{2}}^{1}$, and ${I}_{1\times {S}^{2}}^{2}$ are combined into a single matrix as follows:

10 $${M}_{IRSM}=\left[\begin{array}{c}{I}_{1\times {S}^{2}}\\ {I}_{1\times {S}^{2}}^{1}\\ {I}_{1\times {S}^{2}}^{2}\end{array}\right]=\left[\begin{array}{c}\begin{array}{ccc}\begin{array}{ccc}1& 0& 0\end{array}& \begin{array}{ccc}0& 1& 0\end{array}& \begin{array}{ccc}0& 0& 1\end{array}\end{array}\\ \begin{array}{ccc}\begin{array}{ccc}0& 1& 0\end{array}& \begin{array}{ccc}0& 0& 1\end{array}& \begin{array}{ccc}1& 0& 0\end{array}\end{array}\\ \begin{array}{ccc}\begin{array}{ccc}0& 0& 1\end{array}& \begin{array}{ccc}1& 0& 0\end{array}& \begin{array}{ccc}0& 1& 0\end{array}\end{array}\end{array}\right]$$

In the ${M}_{IRSM}$, the rows correspond to the number of channels ($K$), while the columns represent $N$. Additionally, the number of bits “1” occurrences in each row defines the $W$ parameter. Notably, the cross-correlation between any two code sequences is zero, ensuring the elimination of multiple access interference (MAI) and enhancing system performance. The relation between $K$, $N$, and $W$ is given as:

11 $$N=K W$$

For enhanced security and to leverage the low attenuation properties of the green light spectrum in UOWC systems, wavelengths within the green spectral range are assigned to each channel in ${M}_{IRSM}$. Additionally, three distinct IRSM codewords from ${M}_{IRSM}$ are utilized for transmitting the RGB image separately. Table 2 presents the assigned IRSM codewords for the RGB image, along with their corresponding wavelengths.

**Table 2:** RGB images with corresponding IRSM codewords and wavelengths.

Image	Wavelength (nm)								
	532	532.8	533.6	534.4	535.2	536	536.8	537.6	538.4
IMG 17	1	0	0	0	1	0	0	0	1
IMG 165	0	1	0	0	0	1	1	0	0
IMG 242	0	0	1	1	0	0	0	1	0

### Absorption and scattering coefficients of five types of waterbodies

Light waves interacting with a medium undergo absorption and scattering, which can alter their properties and cause energy loss. In highly turbid water, signal attenuation may render detection making it the main effect to consider in modelling the channel coefficient accurately for proper analysis of wireless optical links^22^. Light propagation depends on absorption and scattering, which determines a medium’s ability to transmit light. Absorption converts light energy into heat, while scattering deflects light due to particles in the medium^23,24^. In UOWC systems, signal transmission can follow either a Line of Sight (LoS) or a Non-Line of Sight (NLoS) configuration^25–27^. Our work focuses on an LoS link where the propagation path loss is expressed according to Beer-Lambert law as^28^:

12 $$PL=\text{exp}\left(-c(\lambda )\frac{L}{\text{cos}{\theta }_{d}}\right)$$

where $PL$, $L$, and ${\theta }_{d}$ are the path loss, the range of the underwater link, and the beam divergence angle, respectively. The extinction coefficient depends on the wavelength and it is represented by $c(\lambda )$ and calculated as the summation of absorption coefficient, $a(\lambda )$, and scattering coefficient, $b(\lambda )$^28^. The coefficient $a(\lambda )$ can be calculated as^15,28^:

13 $$a(\lambda )={\propto }_{PW}\left(\lambda \right) +{\propto }_{Ch}\left(\lambda \right)+{\propto }_{fu}\left(\lambda \right)+{\propto }_{hu}\left(\lambda \right)$$

where ${\propto }_{PW}\left(\lambda \right)$ denotes the absorption coefficient of pure water, the absorption due to chlorophyll is represented by ${\propto }_{Ch}\left(\lambda \right)$ which can be calculated as^15,28^:

14 $${\propto }_{Ch}\left(\lambda \right)=0.0127 {\left(\raisebox{1ex}{$Ch$}\!\left/ \!\raisebox{-1ex}{${Ch}_{0}$}\right.\right)}^{0.62}$$

where $Ch$ is the chlorophyll concentration relative to fixed concentration at 1 mg/${\text{m}}^{3}$ that is given by ${Ch}_{0}$.

Table 3 shows values of $Ch$ of PS, CL, CS, HR I, and HR II at 532 nm^13,29,30^.

**Table 3:** Chlorophyll concentration for five types of waters.

Type of water	* Ch* (mg/m^3^)
PS	0.005
CL	0.31
CS	0.83
HR I	2.99
HR II	5.9

On the other hand, the absorption of fulvic and humic acid are denoted by ${\propto }_{fu}\left(\lambda \right)$ and ${\propto }_{hu}\left(\lambda \right)$. Both are expressed as^13,15,31^:

15 $${\propto }_{fu}\left(\lambda \right)=\left[62.60389982{ C}_{C} \text{exp}\left(\frac{Ch}{{Ch}_{0}}\right)\text{exp}\left(-0.0189\lambda \right)\right]$$

16 $${\propto }_{hu}\left(\lambda \right)=\left[3.64020552{ C}_{C} \text{exp}\left(\frac{Ch}{{Ch}_{0}}\right)\text{exp}\left(-0.01105\lambda \right)\right]$$

The factor $b(\lambda )$ is determined as the linear summation of the scattering coefficients from pure water (${b}_{PW}\left(\lambda \right))$, small particles (${b}_{s}\left(\lambda \right)$), and large particles (${b}_{l}\left(\lambda \right)$), and it is expressed as^13,15,32^:

17 $$b(\lambda )={b}_{PW}\left(\lambda \right) +{b}_{s}\left(\lambda \right)+{b}_{l}\left(\lambda \right)$$

where ${b}_{PW}\left(\lambda \right)$, ${b}_{s}\left(\lambda \right)$ and ${b}_{l}\left(\lambda \right)$ which are calculated as^13,15^:

18 $${b}_{PW}\left(\lambda \right)=0.005826{\left(\raisebox{1ex}{$400$}\!\left/ \!\raisebox{-1ex}{$\lambda $}\right.\right)}^{4.322}$$

19 $${b}_{s}\left(\lambda \right)=1.151302{\left(\raisebox{1ex}{$400$}\!\left/ \!\raisebox{-1ex}{$\lambda $}\right.\right)}^{1.7}\left(0.01739{ C}_{c}\text{ exp} \left[0.11631\left(\raisebox{1ex}{$Ch$}\!\left/ \!\raisebox{-1ex}{${Ch}_{0}$}\right.\right)\right]\right)$$

20 $${b}_{l}\left(\lambda \right)=0.341100{\left(\raisebox{1ex}{$400$}\!\left/ \!\raisebox{-1ex}{$\lambda $}\right.\right)}^{0.3}\left(0.76284{ C}_{C}\text{ exp} \left[0.03092\left(\raisebox{1ex}{$Ch$}\!\left/ \!\raisebox{-1ex}{${Ch}_{0}$}\right.\right)\right]\right)$$

The power of the optical signal after travelling in UOWC is expressed as^13,15,32^:

21 $${S}_{r} ={S}_{t}\cdot {\eta }_{t }\cdot \frac{{A}_{r} cos\varphi }{2\pi {L}^{2}\left[1-cos(\theta )\right]}\cdot {\eta }_{r }\cdot PL$$

where ${S}_{r}$, ${A}_{r}$, and ${\eta }_{rx}$ are received power, receiver aperture area, and optical receiver efficiency, respectively. ${S}_{t}$ and ${\eta }_{t}$ represent transmitted power and optical transmitter efficiency, respectively. The misalignment angle is given by $\varphi$.

In this study, the UOWC channel is modelled as an attenuation-limited medium, where the primary impairments are absorption and scattering. The analysis intentionally neglects the effects of oceanic turbulence and refractive index fluctuations to isolate the deterministic contribution of attenuation in different water types. This assumption enables a clear evaluation of the fundamental performance limits under idealized, turbulence-free conditions. The derived results therefore represent theoretical upper bounds on system performance for each environment, including PS, CL, CS, HR I, and HR II. Such an approach is commonly adopted in foundational analyses, where turbulence is later introduced as a stochastic impairment in subsequent studies to capture real-world variability.

## Proposed secured RGB image transmission in UOWC using IRSM code

**Fig. 1:**
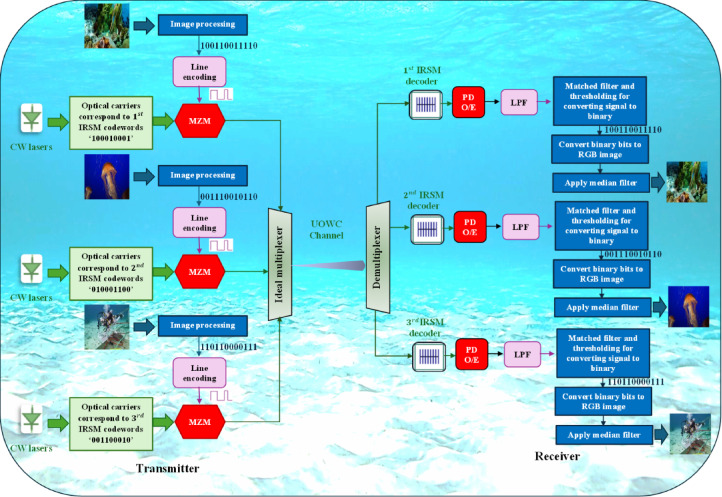
Proposed secured RGB image transmission system in UOWC using IRSM code.

The schematic diagram of the proposed secured RGB image transmission system in UOWC using IRSM code is displayed in Fig. 1. It comprises of the following subsystems.

### Transmitter

The transmitter subsystem used for the three RGB images consists of image processing, an IRSM encoder, line encoding, and modulation. In the image processing stage, each128 × 128 RGB image is converted from pixel values to a binary bit stream $\in \left\{0, 1\right\}$ of length $B$. During line encoding, the binary sequence is modulated using an OOK Non-Return-to-Zero (NRZ) scheme, producing an electrical signal at 10 Gbps. The resulting electrical signal is expressed as^33^:

22 $${E}_{s}(t) =\sum_{i=0}^{B}{a}_{o}\Pi (t-B{T}_{b})$$

where ${a}_{o}$ is the ${i}^{th}$ amplitude symbol, $\Pi (t-B{T}_{b})$ denotes a rectangular pulse shape, and ${T}_{b}$ is the bit duration, defined as $1/{R}_{b}$, where ${R}_{b}$ denotes bit rate.

In the IRSM encoding stage, laser diode (LD) sources generate optical carriers at specific wavelengths assigned according to the IRSM code sequence, as listed in Table 1. The field of the optical wave is given by^34^:

23 $${E}_{op}(t) ={E}_{ampl}{e}^{j({2\pi f}_{LD}t+{\theta }_{LD})}$$

where ${E}_{ampl}$ denotes the amplitude of the optical signal, and ${\theta }_{LD}$ represents the phase shift angle which is set to ${0}^{^\circ }$.

In the modulation stage, a Mach–Zehnder Modulator (MZM) is employed to modulate the electrical OOK-NRZ signal onto optical carriers generated by the LD sources. Although most conventional MZMs are designed for near-infrared wavelengths (around 1550 nm), visible-wavelength LiNbO₃ modulators are both commercially available and an active area of research. Manufacturers such as Exail (formerly iXblue)^35^, and Thorlabs^36^ provide LiNbO₃-based MZMs covering the visible and near-visible spectral ranges (e.g., 635 nm, 780 nm, and 850 nm). Moreover, recent studies have demonstrated the feasibility of thin-film LiNbO₃ MZMs operating efficiently at visible wavelengths, including 473 nm, 520 nm, and 637 nm, with low half-wave voltage and interaction electrode length, confirming their compatibility with green-light communication systems^37^. Thus, the selected MZM operating in the green spectrum is technically feasible and consistent with recent advancements in visible-light modulator design. Finally, the modulated optical carriers from the three transmitters, corresponding to the RGB images, are multiplexed using an ideal multiplexer before propagating through the UOWC channel.

### UOWC channel

The multiplexed optical signal is then transmitted through the UOWC channel. We consider, in this study, five types of water, each characterized by distinct inherent optical properties. The impact of water attenuation, resulting from absorption and scattering coefficients, is discussed in detail in Section “OCDMA code and UOWC channel attenuation”.

### Receiver

The receiver subsystem architecture comprises an IRSM decoder, an Optical-to-Electrical (O/E) converter, an electrical-to-binary converter, an image reconstruction module, a post-processing median filter, and an image quality assessment unit. The IRSM decoder has uniform fiber Bragg gratings (FBGs) that are employed as optical filters, with center wavelengths matching those of the LD sources at the transmitter facilitating the detection of the required RGB image. The detected optical signal is then converted into the electrical domain using a PIN photodetector, followed by a Low-Pass Filter (LPF) to suppress unwanted high-frequency components. The image reconstruction module has a matched filter, which is subsequently applied to optimize signal detection. Next, a decoder circuit determines the binary data by comparing the output signal against a predefined threshold. The recovered binary sequence is then mapped back to pixel values, enabling the reconstruction of the original RGB image. To mitigate the effects of underwater turbulence and signal degradation, a post-processing median filter is applied to enhance visual quality. Finally, the received image quality is assessed using standard IQA metrics.

The proposed system is simulated using Matlab version 2023b and Optisystem version 22 with parameters values given in Table 4^13,14,18^.

**Table 4:** System parameter values.

Image size	$128\times 128$ pixel
${R}_{b}$	10 Gbps
$B$	393,216
CW transmit power	15 dBm
${\eta }_{t}$	0.9
Receiver aperture diameter	20 cm
$\varphi$	${0}^{^\circ }$
${\theta }_{d}$	1 mrad
$L$	Varies according to water type
${\eta }_{r}$	0.9
PIN responsivity	0.8 A/W
LPF cutoff frequency	$0.75\times {R}_{b}$

Although the proposed system is validated through MATLAB and OptiSystem simulations, its experimental realization is feasible using commercially available optical components. There are many published papers in literature that did validate the models of the used component in this work providing confidence about the accuracy of these models in simulating our project. In practical implementation, code generation can be achieved using LD sources, where the emission of a specific wavelength corresponds to bit “1,” and its absence represents bit “0.” At the receiver side, FBG filters can be employed to selectively reflect the encoded wavelengths, enabling accurate decoding of the transmitted code. Moreover, practical deployment would require precise optical alignment, synchronization between the transmitter and receiver, and stabilization of the optical channel to mitigate environmental fluctuations and maintain reliable communication. While minor performance variations are expected between simulation and real-world operation due to optical coupling losses and device tolerances, the overall framework remains technically feasible and scalable for hardware implementation in future UOWC systems.

In this study, the proposed system employs a multi-wavelength OCDMA framework rather than a conventional WDM or single-channel configuration. While WDM can also support parallel multi-wavelength transmission, the OCDMA approach provides additional advantages of enhanced data security and multiple-access capability. Each transmitted wavelength corresponding to the RGB components is uniquely encoded using an IRSM code, which ensures robustness against MAI and mitigates the risk of eavesdropping. Moreover, compared with a single-channel configuration, the proposed system enables true parallel RGB transmission without requiring Time Division Multiplexing (TDM) or compression, thereby maintaining high throughput and spectral efficiency.

## PSO-based image enhancement framework for UOWC

A PSO-driven image enhancement framework is proposed to correct severe image distortions caused by absorption and scattering in UOWC channels. The objective of the PSO algorithm is to optimize adaptively a set of restoration parameters that minimize the perceptual difference between a received distorted image, $R$, and a known original reference transmitted RGB image, $T$. Specifically, a four-dimensional parameter vector $\theta =\left(w, \gamma ,r,d\right)$ is optimized, where $w$ controls Wiener deconvolution, $\gamma$ is the gamma correction exponent, $r$ is the histogram blending ratio, and $d$ determines the wavelet denoising strength.

The PSO algorithm is initiated after executing the SSIM between $R$ and $T$. If the SSIM exceeds 0.95, the image is considered sufficiently accurate and left uncorrected. Otherwise, the PSO process is activated. The PSO algorithm cost function is defined as^38^:

24 $$Cost\left(\theta \right)=1-\frac{1}{3}\sum_{c=1}^{3}\text{SSIM}\left({T}^{c},{R}_{corrected}^{c}\right)$$

where $c$ referes to the color channel index in the image which equals to one for red channel, 2 for green channel, and 3 for blue channel. ${T}^{c}$ denotes $c$-th channel of the original image, and ${R}_{corrected}$ is the corresponding channel of the received image after applying the enhancement pipeline with parameters $\theta$. The cost function evaluates the average $\text{SSIM}$ across all three channels, and its value is minimized during the PSO optimization process.

The restoration pipeline is described below in Algorithm1 and it consists of the following sequential procedures:

i. Dehazing via Underwater Dark Channel Prior (UDCP): A dark channel is applied to suppress haze prior adapting underwater scenes.
ii. Color Compensation: The red channel is boosted to mitigate absorption losses, followed by gray-world white balancing.
iii. Wiener Deconvolution: A Gaussian kernel-based deconvolution is applied to reverse blur induced by scattering.
iv. Gamma Correction: Intensity and contrast are adjusted nonlinearly to match the dynamic range of the reference.
v. Histogram Matching and Blending: Each channel is matched to the reference histogram and blended with the gamma-corrected version.
vi. Wavelet Denoising: Edge-preserving denoising is conducted using Bayesian shrinkage in the wavelet domain.

**Algorithm 1:**
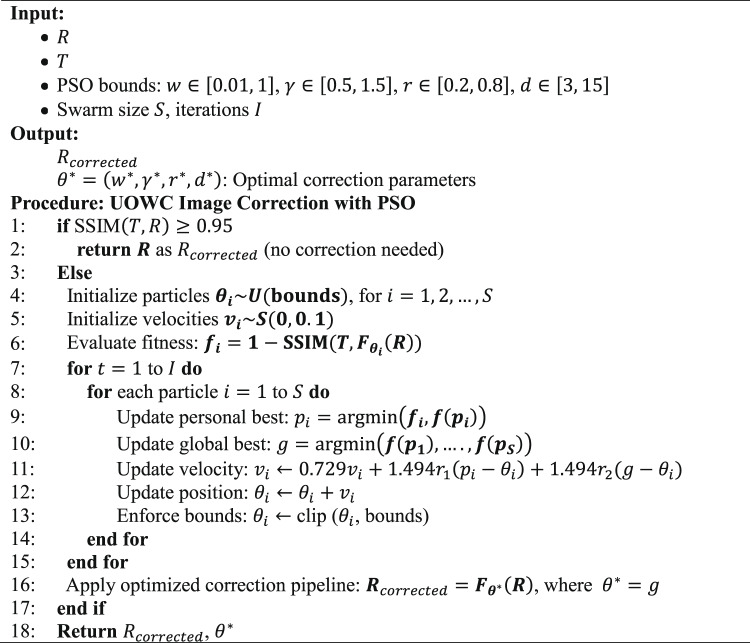
PSO-Based RGB image correction in proposed RGB image transmission system in UOWC using IRSM code.

In the used Algorithm 1, ${F}_{\theta \left(.\right)}$ is the image correction pipeline with parameters $\theta$, incorporating UDCP-based dehazing, red-channel boosting, white balancing, Wiener deconvolution, gamma correction, histogram blending, and wavelet denoising. In this study, The PSO algorithm is executed with a swarm size of 30 particles, a maximum of 50 iterations.

## Simulation results and analysis

This section provides a detailed analysis of the IQA for the three received RGB images assigned with three distinct IRSM codewords after transmission across five different water types. The evaluation is conducted using various metrics, including RMSE, SNR, PSNR, correlation coefficient, SSIM. Each metric is examined to assess the impact of underwater channel conditions on the image fidelity and the transmission performance.

### Impact of Absorption and Scattering Coefficients on RGB Images

**Fig. 2:**
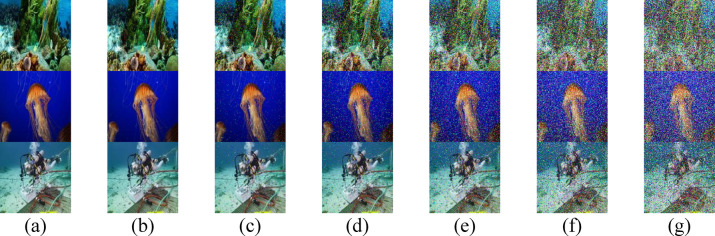
Received images for the proposed secured RGB image transmission in PS water using IRSM code at (**a**) 18 m, (**b**) 19.5 m, (**c**) 21 m, (**d**) 22.5 m, (**e**) 24 m, (**f**) 25.5 m, and (**g**) 27 m. (First row for IMG 17, second row for IMG 165, and third row for IMG 242).

**Fig. 3:**
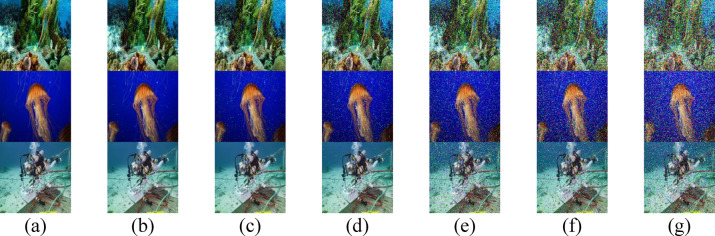
Received images for the proposed secured RGB image transmission in HR II water using IRSM code at (**a**) 3.4 m, (**b**) 3.5 m, (**c**) 3.6 m, (**d**) 3.7 m, (**e**) 3.8 m, (**f**) 3.9 m, and (**g**) 4 m. (First row for IMG 17, second row for IMG 165, and third row for IMG 242).

Both $a(\lambda )$ and $b(\lambda )$ coefficients, which are intrinsic to the optical properties of water, significantly influence the quality of received images in UOWC systems. Higher values of absorption $a(\lambda )$ and $b(\lambda )$ lead to more severe signal degradation, thereby restricting the propagation range. Figures 2, 3 illustrate the quality of three transmitted RGB images (IMG 17, IMG 165, and IMG 242) using three different IRSM codes at various underwater distances across different water types.

**Fig. 4:**
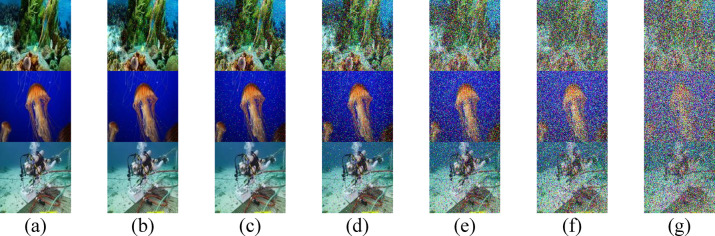
Received images for the proposed secured RGB image transmission in HR I water using IRSM code at (**a**) 5 m, (**b**) 5.2 m, (**c**) 5.4 m, (**d**) 5.6 m, (**e**) 5.8 m, (**f**) 6 m, and (**g**) 6.2 m. (First row for IMG 17, second row for IMG 165, and third row for IMG 242).

**Fig. 5:**
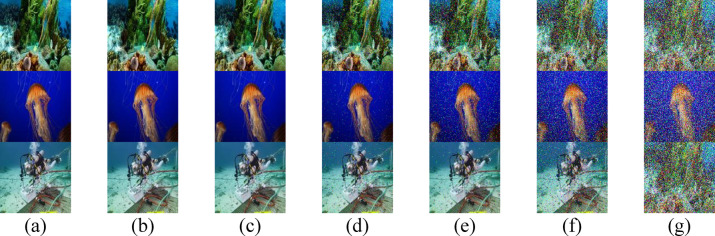
Received images for the proposed secured RGB image transmission in CL water using IRSM code at (**a**) 14 m, (**b**) 14.5 m, (**c**) 15 m, (**d**) 15.5 m, (**e**) 16 m, (**f**) 16.5 m, and (**g**) 17 m. (First row for IMG 17, second row for IMG 165, and third row for IMG 242).

**Fig. 6:**
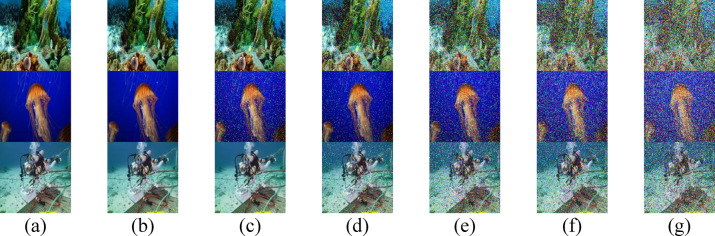
Received images for the proposed secured RGB image transmission in CS water using IRSM code at (**a**) 9 m, (**b**) 9.5 m, (**c**) 10 m, (**d**) 10.5 m, (**e**) 11 m, (**f**) 11.5 m, and (**g**) 12 m. (First row for IMG 17, second row for IMG 165, and third row for IMG 242).

The elevated concentration of $Ch$ in HR I and HR II results in pronounced attenuation, limiting the transmission ranges to 6.2 m and 4 m, respectively, as evident in Figs. 4 and 3. Conversely, PS exhibits the lowest attenuation, allowing the RGB images to achieve the maximum propagation distance of 27 m, as shown in Fig. 2. Additionally, Figs. 5 and 6 demonstrate that CL and CS support moderate transmission distances of 17 m and 12 m, respectively, owing to their attenuation levels being higher than PS but lower than HR I and HR II.

**Fig. 7:**
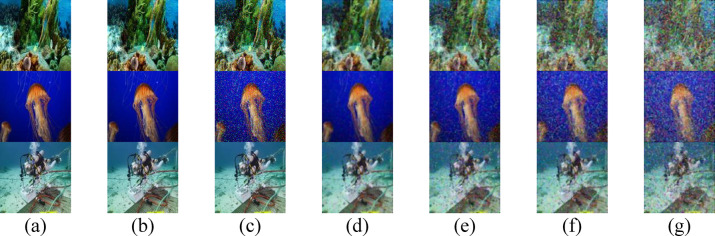
Received images with postprocessing median filter for the proposed secured RGB image transmission in HR II water using IRSM code at (**a**) 3.4 m, (**b**) 3.5 m, (**c**) 3.6 m, (**d**) 3.7 m, (**e**) 3.8 m, (**f**) 3.9 m, and (**g**) 4 m. (First row for IMG 17, second row for IMG 165, and third row for IMG 242).

**Fig. 8:**
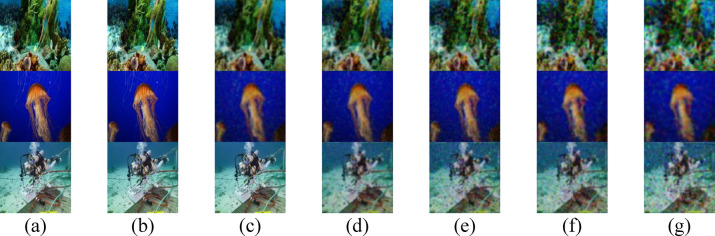
Received images after applying PSO-based correction algorithm for the proposed secured RGB image transmission in HR II water using IRSM code at (**a**) 3.4 m, (**b**) 3.5 m, (**c**) 3.6 m, (**d**) 3.7 m, (**e**) 3.8 m, (**f**) 3.9 m, and (**g**) 4 m. (First row for IMG 17, second row for IMG 165, and third row for IMG 242).

To enhance the visual quality of RGB images, particularly at extended underwater distances, two techniques are employed: median filtering and a PSO-based correction algorithm. The visual quality of the three test images after applying median filtering in HR II water is shown in Fig. 7, while Fig. 8 displays the results after applying the PSO-based correction. A comparative analysis of Figs. 7 and 8 with Fig. 3 reveals that the median filter provides a slight improvement in the perceptual clarity of IMG 17, IMG 165, and IMG 242, particularly at underwater distances ranging from 3.7 to 4.0 m. In contrast, the PSO-based correction algorithm yields more substantial visual enhancement, resulting in noticeably improved image quality across the evaluated distances.

### RMSE of secured RGB images transmission in UOWC system exploiting IRSM code

The RMSE metric evaluates the degree of distortion in the image transmission by calculating the square root of the mean of the squared deviations between the pixel intensities of the original and transmitted images. It can be calculated by^13,39^:

25 $$RMSE =\sqrt{MSE}=\sqrt{\frac{1}{MP}\sum_{x=0}^{M}\sum_{y=0}^{P}{\left(T(x,y)-R(x,y)\right)}^{2}}$$

where the MSE is the mean square error, $M$ and $P$ represent the image dimensions, which are set to 128 in this study. Also, $T$ and $R$ denote the original RGB image before propagation through the UOWC channel and the received RGB image after transmission, respectively. The coordinates $(x,y)$ indicate the pixel position in the image.

**Fig. 9:**
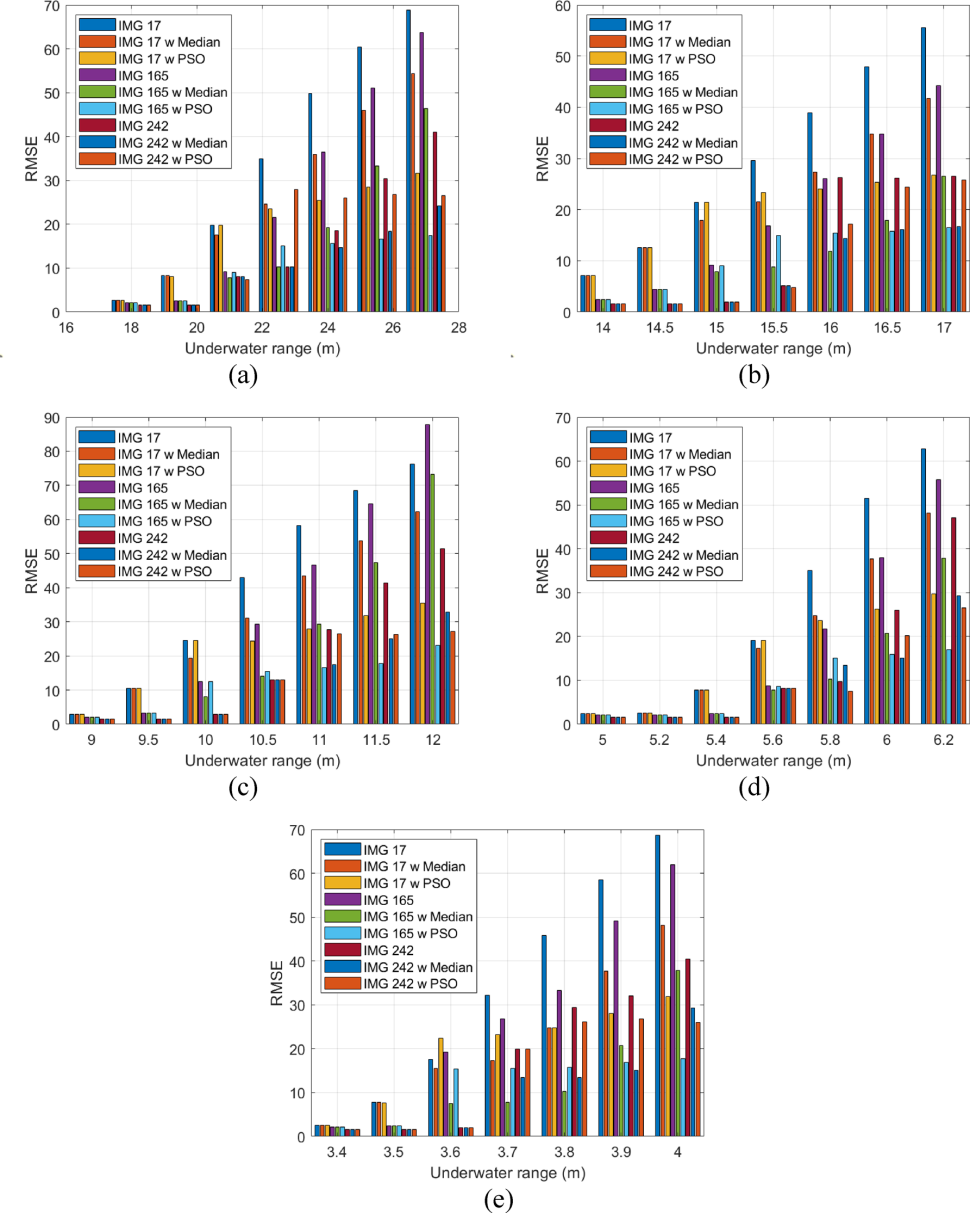
Calculated RMSE for three RGB images assigned with different IRSM codewords for three scenarios versus underwater distances in (**a**) PS, (**b**) CL, (**c**) CS, (**d**) HR I, and (**e**) HR II.

Figure 9 shows the calculated RMSE values for the three investigated RGB images which are transmitted at a data rate of 10 Gbps across five different types of waterbodies, both with and without the application of a median filter. It is evident that RMSE increases as the underwater transmission distance extends across all water types. For instance, as shown in Fig. 9a, IMG 17 exhibits an RMSE of 2.72 for an underwater propagation distance of 18 m in PS. This value soars to 68.8 when the transmission distance extends to 27 m for the same image.

Furthermore, the application of a median filter effectively reduces RMSE for all three images, demonstrating an enhancement in the visual quality. The RMSE values of the IMG 17, IMG 165, and IMG 242 images without the median filter, at a distance of 27 m in PS, 17 m in CL, 12 m in CS, 6.2 m in HR I, and 4 m in HR II are 68.8, 63.67, and 41 (Fig. 9a); 55.51, 44.25, and 25.8 (Fig. 9b); 76.22, 87.84, and 51.46 (Fig. 9c); 62.79, 55.69, and 47.1 (Fig. 9d); and 40.45, 62, and 40.45 (Fig. 9e), respectively.

Conversely, when the median filter is applied, a significant reduction in the RMSE parameter is observed, indicating an improved visual quality in the received images. For instance, the RMSE values for IMG 242 with the median filter at the same distances are 24.11 in PS, 16.7 in CL, 32.91 in CS, 29.26 in HR I, and 29.26 in HR II. These results confirm the effectiveness of using the median filtering in mitigating the distortion and preserving the image fidelity in underwater optical wireless communication. Furthermore, utilizing PSO corrected algorithm enhances the quality of images and reduces the RMSE. Maintaining same range and for the image IMG 242, the RMSE metric is reduced to 26.48, 25.8, 27.27, 26.52, and 25.98, for PS, CL, CS, HR I, and HR II, respectively. It is clear that the use of the PSO-based correction offers a more robust optimization of image restoration parameters, leading to superior perceptual and quantitative quality.

### PSNR and SNR of Secured RGB images transmission in UOWC system with IRSM code

In UOWC systems, the evaluation of image transmission quality and system reliability heavily depends on PSNR and SNR. The PSNR is extracted from the MSE between the original and received images, offering a pixel-based measure of fidelity. Higher PSNR values, typically ranging from 20 to 50 dB for 8-bit images, indicate superior visual quality. It can be expressed as^13,39^:

26 $$PSNR=10{log}_{10}\left(\frac{{Q}^{2}}{MSE}\right)$$

where $Q$ is the maximum pixel value.

Table 4 shows the calculated PSNR for the images IMG 17, IMG 165, and IMG 242 that transmitted on IRSM code-sequences ‘100,010,001’, ‘010001100’, and ‘001100010’, respectively at underwater ranges of 28 m in PS, 17 m in CL, 12 m in CS, 6.2 m in HR I, and 4 m in HR II with and without application of enhancement filter. The utilization of the median filter improves the PSNR values. Moreover, the use of the PSO-based correction algorithm provided an even greater enhancement, yielding higher PSNR values and improved perceptual image quality across various underwater channel conditions as shown in Table 5.

**Table 5:** PSNR for three RGB images transmitted in UOWC and assigned with different IRSM codes.

Water type (range)	Image	PSNR (dB)			Percentage of improvement
		Original	With median filter	With PSO-based corrected	
PS (27 m)	IMG 17	11.37	13.41	18.14	17.94% (median filter) 59.54% (PSO)
	IMG 165	12.05	14.81	23.28	22.90% (median filter) 93.19% (PSO)
	IMG 242	15.87	20.48	19.2	29.05% (median filter) 20.98% (PSO)
CL (17 m)	IMG 17	13.24	15.72	19.57	18.73% (median filter) 47.81% (PSO)
	IMG 165	15.21	19.67	23.82	29.32% (median filter) 56.61% (PSO)
	IMG 242	19.67	23.67	19.89	20.33% (median filter) 1.11% (PSO)
CS (12 m)	IMG 17	10.48	12.23	17.13	16.70% (median filter) 63.45% (PSO)
	IMG 165	9.25	10.83	20.82	17.08% (median filter) 125.08% (PSO)
	IMG 242	13.9	17.78	19.41	27.91%(median filter) 39.64% (PSO)
HR I (6.2 m)	IMG 17	12.17	14.48	18.68	28.98%(median filter) 53.49% (PSO)
	IMG 165	13.21	16.56	23.51	25.36%(median filter) 77.97% (PSO)
	IMG 242	14.66	18.8	19.65	28.24%(median filter) 34.04% (PSO)
HR II (4 m)	IMG 17	11.39	14.48	18.05	27.13%(median filter) 58.47% (PSO)
	IMG 165	12.28	16.56	23.14	34.85%(median filter) 88.44% (PSO)
	IMG 242	15.99	18.8	19.83	17.57%(median filter) 20.01% (PSO)

The SNR is a key metric for assessing the stability of the optical link in UOWC systems. It is defined as the ratio of the total signal power in the original transmitted image to the noise power introduced during transmission. The noise arises due to absorption, scattering, laser, detector and turbulence effects in the underwater channel. Mathematically, the SNR is calculated as^13,39^:

27 $$SNR=10{log}_{10}\left(\frac{\sum_{x=1}^{M}\sum_{y=1}^{P}T{\left(x, y\right)}^{2}}{\sum_{x=1}^{M}\sum_{y=1}^{P}{\left[T(x,y)-R(x,y)\right]}^{2}}\right)$$

Tables 6, 7, 8, 9 and 10 show the SNR for the three RGB images at different underwater transmission ranges for three scenarios (without any enhancement process, with median filter, and with PSO-based corrected algorithm) in PS, CL, CS, HR I, and HR II, respectively. It is clear that when the distance between transmitter and receiver increases, the SNR deteriorates and received RGB images with PSO-based correction at longer underwater distances shows superior in SNR performance.

**Table 6:** SNR for three RGB images transmitted in PS and assigned with different IRSM codes.

Range (m)	SNR (dB)								
	Original			With median filter			With PSO-based correction		
	IMG 17	IMG 165	IMG 242	IMG 17	IMG 165	IMG 242	IMG 17	IMG 165	IMG 242
18	31.02	32.47	38.43	31.02	32.47	38.43	31.03	32.47	38.43
19.5	21.47	30.84	38.43	21.47	30.84	38.43	21.69	30.85	38.43
21	14.29	19.74	24.24	14.92	20.99	24.24	14.29	19.89	25.07
22.5	9.93	12.76	22.26	12.41	18.74	22.26	11.12	14.26	12.84
24	7.44	8.99	17.13	9.64	13.72	19.03	10.59	13.98	12.72
25.5	6.14	6.78	12.86	7.85	9.63	16.99	9.71	13.51	12.45
27	5.27	5.41	10.33	6.66	7.30	14.622	8.96	13.09	11.83

**Table 7:** SNR for three RGB images transmitted in CL and assigned with different IRSM codes.

Range (m)	SNR (dB)								
	Original			With median filter			With PSO-based correction		
	IMG 17	IMG 165	IMG 242	IMG 17	IMG 165	IMG 242	IMG 17	IMG 165	IMG 242
14	22.68	31.35	38.43	22.68	31.35	38.43	22.68	31.34	38.43
14.5	17.97	26.01	38.43	17.97	26.01	38.43	17.97	26.01	38.43
15	13.63	19.73	36.82	14.76	20.99	36.82	13.63	19.89	36.82
15.5	11.16	14.72	28.20	13.41	20.03	28.20	11.18	15.33	28.82
16	9.18	11.36	17.77	11.63	17.57	19.17	10.86	14.08	17.77
16.5	7.73	9.29	14.74	9.88	14.31	18.19	10.56	13.85	14.74
17	6.72	7.70	14.26	8.57	11.30	17.85	10.26	13.51	14.26

**Table 8:** SNR for three RGB images transmitted in CS and assigned with different IRSM codes.

Range (m)	SNR (dB)								
	Original			With median filter			With PSO-based correction		
	IMG 17	IMG 165	IMG 242	IMG 17	IMG 165	IMG 242	IMG 17	IMG 165	IMG 242
9	30.27	32.47	38.43	30.27	32.47	38.43	30.27	32.47	38.43
9.5	19.43	28.61	38.43	19.43	28.61	38.43	19.42	28.61	38.43
10	12.61	17.11	33.07	14.22	20.74	33.07	12.61	17.11	33.07
10.5	8.49	10.52	20.17	10.71	16.18	20.17	10.93	14.02	20.17
11	6.38	7.35	13.66	8.22	10.54	17.50	9.92	13.41	12.51
11.5	5.29	5.33	10.22	6.72	7.15	14.26	8.77	12.81	12.98
12	4.62	3.42	8.37	5.74	4.25	11.80	7.81	10.46	13.12

**Table 9:** SNR for three RGB images transmitted in HR I and assigned with different IRSM codes.

Range (m)	SNR (dB)								
	Original			With median filter			With PSO-based correction		
	IMG 17	IMG 165	IMG 242	IMG 17	IMG 165	IMG 242	IMG 17	IMG 165	IMG 242
5	31.94	32.47	38.43	31.94	32.47	38.43	31.94	32.47	38.43
5.2	31.51	32.47	38.43	31.51	32.47	38.43	31.51	32.47	38.43
5.4	22.07	31.28	38.43	22.07	31.28	38.43	22.07	31.28	38.43
5.6	14.57	20.13	24.20	15.04	20.99	24.20	14.56	20.26	24.20
5.8	9.91	12.73	22.74	12.39	18.77	19.81	11.09	14.22	24.98
6	7.22	8.72	16.37	9.29	13.15	18.75	10.21	13.87	16.37
6.2	5.86	6.24	9.11	7.51	8.70	12.85	9.32	13.25	13.09

**Table 10:** SNR for three RGB images transmitted in HR II and assigned with different IRSM codes.

Range (m)	SNR (dB)								
	Original			With median filter			With PSO-based correction		
	IMG 17	IMG 165	IMG 242	IMG 17	IMG 165	IMG 242	IMG 17	IMG 165	IMG 242
3.4	31.46	32.47	38.43	31.46	32.47	38.43	31.46	32.47	38.43
3.5	22.05	31.28	38.43	22.05	31.28	38.43	22.16	31.28	38.43
3.6	15.23	13.65	36.70	15.76	21.36	36.70	11.45	14.12	36.70
3.7	10.55	11.16	16.46	15.04	20.99	19.83	11.25	13.89	16.46
3.8	8.00	9.63	13.15	12.39	18.77	19.81	10.81	13.85	13.05
3.9	6.32	7.03	12.40	9.29	13.15	18.75	9.85	13.33	12.71
4	5.28	5.56	10.42	7.51	8.70	12.85	8.80	12.77	12.47

### SSIM of secured RGB images transmission in UOWC system with IRSM code

Unlike conventional pixel-level metrics such as PSNR, the SSIM evaluates the structural distortions by analyzing luminance, contrast, and texture differences between the original and reconstructed images. This approach provides a more perceptually relevant assessment aligning more closely with human visual perception. It has values spans from -1 to 1, where a value of 1 signifies perfect similarity between images. It can be computed as^13,39^:

28 $$SSIM(T,R)=\frac{(2{\mu }_{T}{\mu }_{R}+{C}_{1})\left(2{\sigma }_{TR}+{C}_{2}\right)}{\left({\mu }_{T}^{2}+{\mu }_{R}^{2}+{C}_{1}\right)\left({\sigma }_{T}^{2}+{\sigma }_{R}^{2}+{C}_{2}\right)}$$

where ${\mu }_{T}$ and ${\mu }_{R}$ denote the mean pixel values of the transmitted and received images, respectively, and ${\sigma }_{TR}$ represents their covariance. To prevent numerical instability when the means or variances approach zero, small constants ${C}_{1}$ and ${C}_{2}$ are introduced, set to ${(0.01\times 127)}^{2}$ and ${(0.03\times 127)}^{2}$, respectively.

**Fig. 10:**
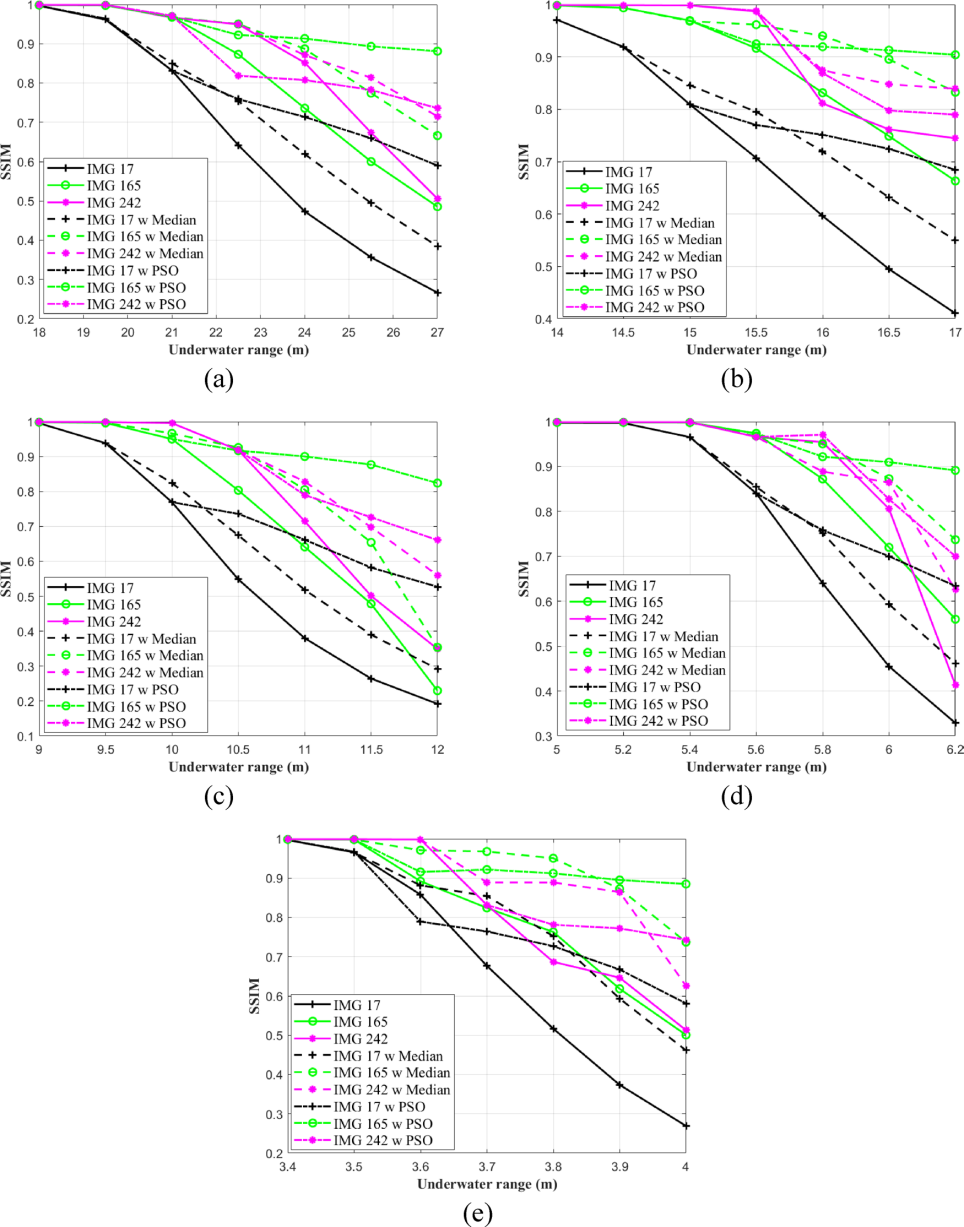
Calculated SSIM for three RGB images assigned with different IRSM codewords versus underwater distances in (**a**) PS, (**b**) CL, (**c**) CS, (**d**) HR I, and (**e**) HR II.

Figure 10 presents the calculated SSIM values between the transmitted and received RGB images for five different water types, with and without the application of a median filter. It is observed that as the underwater transmission distance increases, the SSIM values decrease. Additionally, a slight improvement in the SSIM values is noted when a median filter is applied.

Figure 10a displays SSIM at an underwater distance of 27 m in PS, the SSIM values for IMG 17, IMG 165, and IMG 242 are 0.26, 0.48, and 0.50, respectively. The application of the median filter enhances these values to 46.15% for IMG 17, 37.5% for IMG 165, and 42% for IMG 242, reaching 0.38, 0.66, and 0.71, respectively. In the case of CL and CS, as shown in Fig. 10b and c, the IMG 165 exhibits SSIM values of 0.66 at 17 m in CL and 0.22 at 12 m in CS. When the median filter is applied, the SSIM values improve to 0.54 and 0.35 for the same respective distances. For harbor waters (HR I and HR II), the SSIM values exceeded 0.5 for IMG 17, IMG 165, and IMG 242 at underwater distances of 5.8 m and 3.8 m, 6.2 m and 4.0 m, and 6.0 m and 4.0 m, respectively, as depicted in Fig. 10d and e. Furthermore, applying the median filter enables all images to achieve SSIM values close to or exceeding 0.5 at an underwater transmission range of 6.2 m in HR I and 4.0 m in HR II. In contrast, the PSO-based correction algorithm yields the highest SSIM values for all images, indicating superior perceptual quality. Specifically, in HR II at a transmission distance of 4.0 m, the SSIM values for IMG 17, IMG 165, and IMG 242 are 0.58, 0.88, and 0.74, respectively.

### Correlation coefficient of secured RGB images transmission in UOWC system with IRSM code

The correlation coefficient, denoted as $\text{CF}$, measures the strength of the linear relationship between two images, serving as an additional metric for assessing their similarity. This parameter is frequently utilized in image processing to quantify the degree of resemblance between images. The value of $\text{CF}$ ranges from − 1 to 1, where a value of $\text{CF}$ equals to 1 indicates a perfect positive correlation (identical images), while a value of $-1$ signifies a complete negative correlation (images are exact inverses), and a value of $0$ implies no linear correlation. The $\text{CF}$ is mathematically represented as^13,39^:

29 $$CF(T,R)=\frac{\sum_{x=1}^{M}\sum_{y=1}^{P}(T(x,y) -{\mu }_{T})(R(x,y)-{\mu }_{R})}{\sum_{x=1}^{M}\sum_{y=1}^{P}{\left(T(x,y) -{\mu }_{T}\right)}^{2}\sqrt{\sum_{x=1}^{M}\sum_{y=1}^{P}{\left(Y(x,y)-{\mu }_{R}\right)}^{2}}}$$

**Fig. 11:**
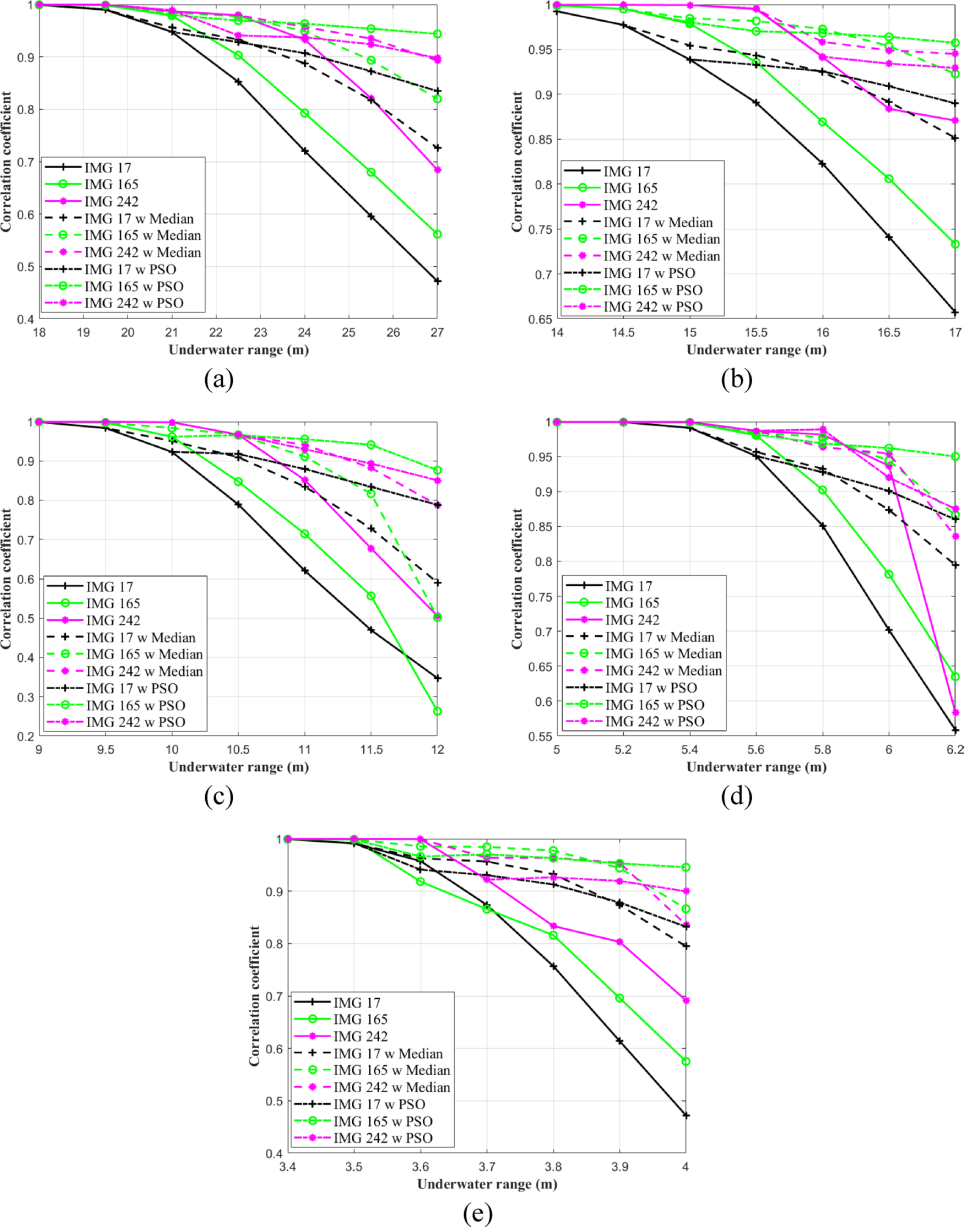
Calculated $\text{CF}$ for three RGB images assigned with different IRSM codewords versus underwater distances in (**a**) PS, (**b**) CL, (**c**) CS, (**d**) HR I, and (**e**) HR II.

Figure 11 presents the calculated $\text{CF}$ values for three RGB images, each encoded with distinct IRSM codes, across varying underwater distances in PS, CL, CS, HR I, and HR II water types. As observed in Fig. 11a for PS water conditions, the $\text{CF}$ remains close to 1 at an underwater distance of 18 m. However, as the transmission range extends to 27 m, the $\text{CF}$ decreases to 0.47 for IMG 17, 0.56 for IMG 165, and 0.68 for IMG 242 when no enhancement is applied. The incorporation of post-filters significantly improves the $\text{CF}$ metric. For instance, at a transmission distance of 27 m, the CF for IMG 17 increases from 0.47 to 0.72 following the application of the median filter. Furthermore, under the same underwater conditions and for the same image, the CF increases to 0.83, which corresponds to a 76.59% improvement when the PSO-based correction algorithm is applied. A similar enhancement is observed across other water types, particularly at longer underwater distances, as illustrated in Figs. 10b–e.

Table 11 shows the number of bits “1 s” and “0 s” for the three investigated RGB images.

**Table 11:** Binary distribution of bits for the three RGB images.

Image	Total number of bits	Total number of bit “1”	Total number of bit “0”
IMG 17	393,216	161,719	231,497
IMG 165	393,216	137,119	256,097
IMG 242	393,216	194,533	198,683

Further analysis indicates that the RGB image with a higher proportion of binary ones are more vulnerable to signal attenuation in UOWC systems due to the medium’s absorption and scattering characteristics. This binary imbalance, especially prevalent in brighter image regions, leads to a more significant signal degradation during transmission. In such scenarios, median filtering consistently provides superior performance over the particle PSO-based image correction algorithm in terms of SNR and PSNR. The median filter excels at reducing noise while maintaining critical image details such as edges and textures. On the other hand, the PSO-based method, despite its adaptive nature, may struggle to converge on optimal correction parameters when the image distortions result from complex propagation conditions. These findings underscore the effectiveness of median filtering for enhancing image quality in high-speed UOWC systems, particularly when transmitting images with skewed binary bit distributions.

Additionally, Table 12 is added at the end of subsection “Correlation coefficient of secured RGB images transmission in UOWC system with IRSM code” in the revised manuscript as follows:

**Table 12:** Comparison between PSO-corrected algorithm and median + CLAHE technique for enhancing received 165 images in HR II water.

Range	PSO				Median + CLAHE			
	PSNR	SNR	SSIM	CF	PSNR	SNR	SSIM	CF
3.4	41.73	32.47	1.00	1.00	18.07	10.63	0.86	0.94
3.5	40.53	31.28	1.00	1.00	18.30	10.80	0.86	0.94
3.6	24.39	14.12	0.92	0.97	15.85	8.97	0.79	0.92
3.7	24.30	13.89	0.92	0.97	17.46	10.14	0.83	0.94
3.8	24.17	13.86	0.91	0.96	14.73	8.17	0.75	0.90
3.9	23.61	13.33	0.90	0.95	11.54	6.05	0.63	0.82
4	23.14	12.77	0.88	0.95	9.54	4.83	0.54	0.71

To further validate the effectiveness of the proposed PSO-based image enhancement technique, a comparative benchmarking analysis is conducted against the widely used Contrast Limited Adaptive Histogram Equalization (CLAHE) method. Table 12 presents the PSNR, SNR, SSIM, and CF metrics for received RGB image 165 in HR II water, comparing the PSO-corrected results with those obtained using the Median + CLAHE approach. The results demonstrate that PSO consistently achieves higher PSNR, SNR, and SSIM values across all tested ranges, with CF approaching unity, confirming its superior performance in restoring underwater distorted images. Although this comparison is performed for one representative RGB image under HR II conditions, it establishes a clear performance benchmark for the proposed optimization-based approach.

### Computation complexity analysis for using PSO correction

The computational complexity of the proposed PSO-based image correction framework is determined by the number of operations required to optimize and apply the enhancement pipeline to each received image. The process involves iterative evaluation of a perceptual cost function that includes SSIM-based similarity measurements and pixel-wise image correction across all RGB channels. Given the nature of evolutionary optimization, the total complexity scales with the number of particles in the swarm, the number of PSO iterations, and the spatial resolution of the image. Specifically, each particle performs a complete enhancement of the distorted image and evaluates its similarity to the reference image, leading to a cumulative cost that increases linearly with the swarm size and number of iterations. The following analysis outlines the per-image computational load in terms of key algorithmic parameters and quantifies the total number of operations for typical system settings. Table 13 summarizes the runtime performance of the proposed PSO-based image correction.

**Table 13:** Runtime performance comparison for PSO algorithm.

Parameter	Value
System configuration	Intel Core i9-12900H, 20 threads, 32 GB RAM
Image size	128 × 128
Swarm size	30
Iterations	50
Average runtime per image	41.32 s

Although the average runtime of approximately 41.32 s per 128 × 128 RGB image indicates non-real-time performance, the results confirm the algorithm’s capability to substantially enhance image perceptual quality and quantitative IQA metrics. In real-world IoUT deployments, significant acceleration can be achieved through GPU-based parallelization or hardware implementation on FPGAs and embedded GPUs (e.g., NVIDIA Jetson). Moreover, employing a hybrid learning model that approximates PSO-optimized parameters can enable near real-time inference while improving energy efficiency and scalability in practical UOWC systems.

Table 14 presents the computational complexity breakdown highlighting the scalability of the method with respect to image size, swarm parameters, and pipeline depth.

**Table 14:** Computational complexity analysis for PSO algorithm.

Component	Complexity	Complexity value	Description
Input image size	$M\cdot P \cdot c$	49,152	Per-pixel operations across all color channels
Image enhancement per particle	$E(MPc$)	49,152	Full pipeline: dehazing, correction, denoising
SSIM computation per particle	$E(MPc$)	49,152	SSIM per channel
Cost evaluation per iteration	$E(S\cdot MPc$)	1,474,560	Each particle evaluated once per iteration
Total PSO optimization (per image)	$E(I\cdot S\cdot MPc$)	73,728,000	Total enhancement complexity

The computational complexity of the proposed PSO-based framework scales linearly with image resolution since enhancement and SSIM computations are performed per pixel across RGB channels. Increasing the image size from 128 × 128 to 512 × 512 (16 × more pixels) would proportionally raise the total operation count. Although this leads to higher computational demand, a real-time performance can be achieved through GPU-based parallelization or hardware acceleration on FPGAs and embedded GPUs. Additionally, adaptive-resolution or region-based processing can reduce the computational load while preserving visual fidelity, ensuring scalability and energy-efficient operation for IoUT applications.

## Conclusion

In this paper, we proposed a secure image transmission system using IRSM codes to enhance and secure UOWC systems. Three different images (IMG 17, IMG 165, and IMG 242) are assigned distinct IRSM codewords, ensuring that only receivers with knowledge of the code can recover the transmitted RGB images. We analyzed the impact of absorption and scattering coefficients of five different water types on the quality of RGB images at various propagation ranges. Image quality is evaluated using well-known IQA metrics, including RMSE, PSNR, SNR, SSIM, and CF. To mitigate the impact of water attenuation, which introduces noise in the received images, we applied a post-processing median filter.

Our results show that propagation range in underwater environments is limited by water type. For example, PS supports the longest transmission distance of up to 27 m due to lower attenuation. In contrast, RGB images achieve moderate transmission spans of 17 m in CL and 12 m in CS, as they exhibit higher attenuation than PS. Harbor waters, which have the highest chlorophyll concentration, result in the shortest transmission ranges of 6.2 m (HR I) and 4 m (HR II). Additionally, the IQA metrics deteriorate as underwater distance increases. For instance, at an underwater link of 3.4 m, IMG 17 has RMSE = 2.59, PSNR = 39.84 dB, SNR = 31.45 dB, SSIM = 0.99, and CF = 0.99. These values degrade to RMSE = 68.65, PSNR = 11.39 dB, SNR = 5.27 dB, SSIM = 0.26, and CF = 0.47 when IMG 17 is transmitted over 4 m.

Moreover, post-processing filtering enhances the visual quality of received RGB images, particularly at longer transmission ranges. Furthermore, applying the PSO-based correction algorithm shows a notable improvement in image quality. This enhancement is attributed to the algorithm’s ability to adaptively optimize multiple restoration parameters such as deblurring strength, gamma correction, histogram alignment, and denoising based on the structural similarity with a reference image. As a result, the correction process becomes more tailored to the specific distortion characteristics introduced by the underwater optical channel, leading to superior perceptual fidelity and improved quantitative metrics such as CF, SSIM, and PSNR. Each image is transmitted at 10 Gbps, leading to a total system capacity of 30 Gbps, making it suitable for marine applications such as the Internet of Underwater Things (IoUT), oil exploration, and oceanographic research. Additionally, the use of IRSM codes enhances security, making the proposed system valuable for military applications in underwater environments.

In future work, the analysis could be extended to include temperature and salinity-induced turbulence, as well as other real-world challenges such as pointing errors, node mobility, background light interference, and energy-efficient transmission design to enhance system robustness and sustainability in practical UOWC deployments.

## Data Availability

The data used and/or analyzed during the current study are available from the corresponding author on reasonable request.
